# Physico-Chemical, Textural and Sensory Evaluation of Spelt Muffins Supplemented with Apple Powder Enriched with Sugar Beet Molasses

**DOI:** 10.3390/foods11121750

**Published:** 2022-06-14

**Authors:** Biljana Lončar, Lato Pezo, Vladimir Filipović, Milica Nićetin, Jelena Filipović, Milada Pezo, Danijela Šuput, Milica Aćimović

**Affiliations:** 1 Faculty of Technology Novi Sad, University of Novi Sad, Bulevar Cara Lazara 1, 21000 Novi Sad, Serbia; vladaf@uns.ac.rs (V.F.); milican@uns.ac.rs (M.N.); suput.danijela@gmail.com (D.Š.); 2 Institute of General and Physical Chemistry, University of Belgrade, 11000 Belgrade, Serbia; latopezo@yahoo.co.uk; 3 Institute of Food Technology, University of Novi Sad, Bulevar Cara Lazara 1, 21000 Novi Sad, Serbia; jelena.filipovic@fins.uns.ac.rs; 4 Department of Thermal Engineering and Energy, “Vinča” Institute of Nuclear Sciences—National Institute of the Republic of Serbia, University of Belgrade, P.O. Box 522, 11001 Belgrade, Serbia; milada@vin.bg.ac.rs; 5 Institute of Field and Vegetable Crops Novi Sad—National Institute of the Republic of Serbia, Maksima Gorkog 30, 21000 Novi Sad, Serbia; acimovicbabicmilica@gmail.com

**Keywords:** artificial neural network, molasses, supplement apple powder, spelt muffins, principal component analysis

## Abstract

The present study investigated the effect of incorporating 10, 20, and 30% apple powder obtained by freeze-drying, and apple powder produced with osmotic pre-treatment in sugar beet molasses solution, into muffins. The powder was freeze-dried and introduced as a whole spelt wheat flour replacement in muffins. The obtained products were investigated for their chemical composition and technological properties, and were subjected to a sensory analysis as well as a consumer acceptance test. Increasing the substitution level from 0 to 30% apple powder lowered the protein, starch, and fat content, while moisture content, sugar, and cellulose showed the opposite trend. The sensory analysis results indicated that the addition of apple powder or apple powder with osmotic pre-treatment (apple OT+Lyo powder) to the ingredients of muffins positively affected the taste, smell, mastication, and appearance of the final product. Consumers rated the muffins with 30% apple OT+Lyo powder as the most acceptable. Principal component analysis, an artificial neural network, and global sensitivity analysis were utilized to differentiate among muffin samples, and to estimate the corresponding influence of the substitution of spelt flour with apple powder or apple OT powder on the observed quality and nutritional parameters of the muffins.

## 1. Introduction

Muffins are favored for breakfast and snack food due to their ready-to-eat form, low price, tender texture, and sugary taste [1,2]. The primary ingredients for muffin batter (wheat flour, oil, egg, sugar, and milk) significantly influence the muffins’ structure, appearance, and quality [3,4]. This kind of bakery product is often rich in sugar and fat, while deficient in dietary fibre, antioxidants, and minerals [5]. Recent research developed a vegan muffin using the powdered cooking water of yellow soybeans instead of egg whites [6]. The quality of muffins can be easily upgraded to fulfill specific dietary requirements using fruit-based functional ingredients that can enhance muffin properties [7,8,9]. Changing the traditional composition of batter may change the sensory properties and consumer acceptance of muffins regarding color, texture, and flavor [10]. The desirability of the baked products is immensely affected by the quality and balance of the recipe [11]. A multi-disciplinary strategy should be employed in creating novel baked goods that are well received by consumers, evaluating several factors at once, including the nutritional profile and sensory and technological characteristics, in order to develop new products that provide satisfactory taste [12].

Fibre (fruit or cereal source) can be added into baked products as a replacement for traditional ingredients such as flour, sugar, or fat [13,14]. In addition, fruit fibre is beneficial for health as a result of its unique functional properties, including high total and soluble fibre content, water and oil holding capacity, colonic fermentability, and lower phytic acid content and caloric value [15]. The addition of dietary fibre into baked products is related to higher batter viscosity, which affects product volume and texture [16]. A wealth of research [17,18,19,20,21] into apples emphasizes their significance in the healthy human diet due to their high levels of flavonoids, ascorbic acid, pectin, and their low content of sodium, fat, and calories [22].

The application of osmotic treatment (OT) as a pre-treatment for fruit powder production enhances the powder’s quality, nutrient composition, and energy efficiency [23]. Furthermore, an essential advantage of sugar beet molasses utilization as a hypertonic medium for OT, besides efficient water removal, is that it enriches the food material with nutrients that infiltrate from the molasses into the plant tissue [24,25].

Spelt wheat is an old subspecies of traditional wheat with technological properties that are slightly diverse from common wheat, and is usually viewed as a flawed grain [26]. Nevertheless, spelt wheat has been incorporated into different foods, such as bread, pasta, breakfast cereal, and other products with modified nutritional properties. Spelt incorporates enhanced levels of nutrients into food compared to conventional wheat products [27]. Spelt-based products are possibly more digestible and therapeutically beneficial (prevention of cancer and cardiovascular disease, favorable effects on the immune system and blood circulation, reduction in blood cholesterol levelsblood, allergy relief) than products obtained from conventional wheat [28].

Using principal component analysis (PCA) enables differentiation among the investigated muffin samples and a graphical presentation of relations between observed parameters. The effects of substituting flour with apple powder and OT apple powder on the chemical composition, technological characteristics, and color of muffins was examined using global sensitivity analyses based on the developed ANN model. Artificial neural networks (ANN) in diverse implementations usefully contribute to the engineering, technological processes, and development of food products [29].

The present study focused on the exploitation of apple powder prepared by freeze-drying (Lyo apple powder) and apple powder obtained with osmotic pre-treatment in sugar beet molasses and freeze-drying (OT+Lyo apple powder), in varying levels (10%, 20%, and 30%), as a functional ingredient in whole spelt wheat muffins. In addition, the effects of both apple powder varieties’ incorporation on muffins’ chemical composition and technological and sensorial properties were studied. A consumer acceptance test was performed in order to select the muffins with the best properties.

## 2. Materials and Methods

### 2.1. Apple Powder Preparation

Granny Smith apples were acquired at a nearby market in Novi Sad (Serbia) and kept at 4 °C before use. This apple variety was selected due to its acidic, sharp, and juicy tart flavor, which provides enhanced sensory attributes to the muffin samples. The initial moisture content was 85.48%. The osmotic treatment was performed in sugar beet molasses (pH 8.2 and 80% dry matter), as described by Nićetin et al., 2017 [30]. Water loss (WL) and solid gain (SG) were estimated as specified by Koprivica et al., 2014 [31]. After the osmotic treatment, apple samples (WL 55.257g/g_in.s_, SG 2.624g/g_in.s._) were stored in a freezer at −18 °C for 24h. The frozen apples were freeze-dried using a freeze dryer (Alpha 1-2 LD plus CHRIST, Martin Christ Gefriertrocknungs anlagen GmbH, Osterode am Harz, Germany). The samples were placed on metal trays, and the freeze-drying parameters were set to a pressure of 1.6 Pa and condenser temperature of −57 °C. The FD process was performed as described by Nowak and Jakubczyk, 2020 [31].

The Lyo apple powder was obtained by freeze-drying, while OT+Lyo apple powder was obtained via the hybrid drying method. For preparation of OT+Lyo apple powder, the apple samples were dried applying a freeze drier (FD) and coupling this action with osmotic treatment of sugar beet molasses prior to the FD step; this was performed until the final moisture content was 12.03% (*w*/*w*). Dried samples were finely ground into a powder, using a universal laboratory mill type WZ-1 (Solem, ZBPP, Bydgoszcz, Poland).

### 2.2. Muffin Preparation

Muffins were prepared following the adjusted method presented by Nicol [32]. The batter was based on commercially available ingredients—120 g of spelt whole flour (obtained from “Lučar d.o.o.”, Novi Sad, Serbia), 60 g of sugar (“Šajkaška” Žabalj, Serbia), 20 g of sunflower oil (“AD Dijamant”, Zrenjanin, Serbia), 50 g of skimmed milk (“Imlek” Belgrade, Serbia), 50 g of eggs, 2 g of baking powder (“Dr. Oetker” Šimanovci, Serbia), 0.4 g of salt (“SO Produkt”, Stara Pazova, Serbia), and 1 g of cinnamon (“Dr. Oetker” Šimanovci, Serbia). Sugar, eggs, oil, and milk were blended for 2 min using a two-pin mixer (Bosh series, Slovenia). Spelt whole flour, apple powder, baking powder, and cinnamon were then added and mixed for 20 s. The batter (50 g) was poured into a muffin cup and baked at 200 °C for 25 min in a preheated oven. Muffins were prepared in 6 variants (plus control) that differed in spelt whole flour substitution with the apple powder and OT apple powder, in proportions of 10, 20, and 30%. The muffin formulation that contained only spelt whole flour was taken as the control sample (Table 1).

**Figure 1:**
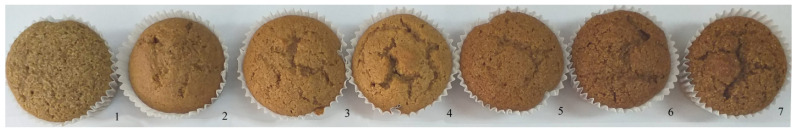
Muffin samples with Lyo apple powder and OT+Lyo apple powder. Samples are numbered according to Table 1.

Figure 1 visually presents muffin samples after preparation. The samples are listed according to Table 1. The change in muffin samples’ color and texture with the addition of apple and OT apple powder is noticeable from Figure 1.

### 2.3. Proximate Composition

The muffin samples were analysed for their protein, moisture, starch, fat, cellulose, and sugar content by applying the standard methods of analysis [33]. The contents of Ca, Mg, K, and Na were obtained by utilizing an Atomic Absorption Spectrophotometer [34] (method No. 984.27) on a Varian Spectra AA 10 (Varian Techtron Pty Ltd., Melbourne, Victoria, Australia). Each measurement was replicated three times.

### 2.4. Technological Characteristics

Technological characteristics (specific volume, specific weight, hardness, and springiness) of muffins with Lyo apple and OT+Lyo apple powder were analysed.

Each muffin sample was measured for its specific weight and specific volume 24 h after baking, according to the AACC method [34]. A Scanner Volscan Profiler (Stable Micro Systems, Godalming, UK) was used for specific volume measurement. Data were presented as the means of three measurements.

The texture of the muffins was determined instrumentally on a texture analyzer TA.XTplus (Stable Micro Systems, Godalming, UK). The hardness and springiness of the muffins were measured using a 36-millimetre cylinder probe (P/36R) and a 5-kilogram load cell. Instrument settings were as follows: pre-test speed, test speed, and post-test speed were 2.0 mm/s, 1.0 mm/s, and 10.0 mm/s, respectively. The muffins were sliced off the top crust in order to obtain the soft inside of the muffin, and eachmuffin was perforated for testing. The muffins were evaluated at 25% compression strain. Hardness (g) was defined as the force required to compress the product by a present distance of 25%. A simple way of looking at the springiness property is to record the force after 30 s, divide this by the maximum force and multiply by 100%. The tests were performed using six replicates per batch.

### 2.5. Muffin Color

The muffin color was measured using a tri-stimulus color meter type CR-400 (Konica, Minolta, Tokyo, Japan) equipped with D65 illuminant. The results were expressed as per CIELab system in terms of coordinates: L*—lightness (0, black to 100, white), a*—redness (−a*, green to +a*, red), and b*—yellowness (−b*, blue to +b*, yellow); W—whiteness is an attribute by which an object is judged to approach the preferred white. The measurements were observed under constant lighting conditions, at 28 °C, using the color attributes of a white control plate, L* = 98.76, a* = −0.04, and b* = 2.01 [35].

Colour variations between the control muffin sample and muffin samples with apple powder and OT apple powder (ΔE), were determined by the following equation:

(1) $$\Delta E=\sqrt{{\Delta L}^{2}+{\Delta a}^{2}+{\Delta b}^{2}}$$

where the variables are defined as follows:

ΔL*—difference in L* parameter between control and muffin sample with apple powder addition;
Δa*—difference in a* parameter between control and muffin sample with apple powder addition;
Δb*—difference in b* parameter between control and muffin sample with apple powder addition.

### 2.6. Sensory Evaluation of Muffins

Sensory evaluation of muffins with apple and osmotically treated apple powder was conducted as described by Filipović et al. 2016 [36] and ISO 4121:2002 [37], by an expert team of 10 qualified evaluators. The sensory analysis was completed at the Institute of Food Technology Novi Sad, Novi Sad, Serbia, in a sensory testing laboratory. Distilled water was used to rinse the mouth between muffin samples during the evaluation [37,38]. A methodology based on the identification of appropriate descriptors was applied in order to assess sensory properties. A list of four sensory descriptors (smell, taste, appearance, and mastication) has been established, the intensity of which is expressed using a linear scale from 0 to 5, (where 0 = unacceptable, 1 = bad, 2 = acceptable, 3 = good, 4 = very good, 5 = excellent quality).

### 2.7. Consumer Acceptance Test

With the intention of testing the consumer acceptability of muffins with apple and OT apple powder, a total of 250 untrained assessors were engaged in this examination. All respondents have consented to participation in the study. Consumers performed the test in order to assess the quality and acceptability of muffins. Sensory parameters (taste, smell, chewing behavior, and general acceptability) of the muffins with the addition of apple powder and osmotically treated apple powder were rated on a 9-level hedonic scale (1 = extremely disliked it, 2 = very disliked it, 3 = moderately disliked it, 4 = disliked it slightly, 5 = neither like nor dislike, 6 = liked it slightly, 7 = liked it moderately, 8 = liked it very much, 9 = liked it extremely) [39].

### 2.8. Statistical Analysis

Descriptive statistical analyses using one-way ANOVA of the results (displayed as the mean ± standard deviation—SD) were conducted with StatSoft Statistica 10.0 software. The significant differences were resolved according to the post hoc Tukey’s HSD (honestly significant differences) test at *p* ≤ 0.05 significance levels. PCA analyses, an ANN model, global sensitivity analysis, and correspondence analysis of the acquired results were conducted by applying StatSoft Statistica 10.0^®^ software.

The color plot diagram was designed with R software v.4.0.3 (64-bit version) with the “circle” method, upper type.

#### Global Sensitivity Analysis

Yoon’s global sensitivity formula for the obtained ANN model was exploited in order to evaluate the relative influence of the input parameters on output variables, based on weight coefficients of the established ANN models [40]:

(2) $$RI_{g}\left(\%\right)=\frac{\sum_{k=0}^{n}\left(w_{ik}\cdot w_{kj}\right)}{\sum_{i=0}^{m}\left|\sum_{k=0}^{n}\left(w_{ik}\cdot w_{kj}\right)\right|}\cdot 100\%$$

where: *w*—weight coefficient in ANN models, *i*—input variable, *j*—output variable, *k*—hidden neuron, *n*—number of hidden neurons, *m*—number of inputs.

## 3. Results and Discussion

The addition of Lyo apple and OT+Lyo apple powder changes the chemical composition of muffins (Table 2). The addition of Lyo apple powder and osmotically treated (OT) apple powder in the amounts of 10%, 20%, and 30% in the muffins leads to a statistically significant enhancement in the moisture content of the finished product (samples 2–4 and samples 5–7); this shows that the samples with apple have a minor percentage of dry matter compared to raw material composition of the control sample (1). With augmentation of the added content of Lyo apple powder (10%, 20%, 30%) and osmotically pre-treated Lyo apple powder (10%, 20%, 30%) in the muffin, there is a statistically significant decrease in protein, starch, and fat, and a statistically significant increase in cellulose and sugar (samples 3, 4, 6, and 7) compared to the control sample (1). Previous studies identified that these kinds of fruit powders raised the baked products’ fibre content and nutritional values [10]. The reduction in protein and starch content and the increase in cellulose and sugar in muffins (samples 2–7) is the reason why flour is replaced with a non-starch and non-protein component, i.e., apple [41]. Intake of food enriched with fruits and vegetable powder in the form of functional products effectively decreases the risk of various diseases, including cancers, diabetes, atherosclerosis, and ulcers [42].

The substitution of flour with Lyo apple and osmotically pre-treated Lyo apple powder affects the mineral content of muffins (Table 3). By increasing the amount of Lyo apple powder (10%, 20%, 30%), there is a corresponding increase in Ca and Na in muffins (sample 2, 3, 4), along with a statistically significantly decrease in Mg and K (sample 2, 3, 4) compared with control sample 1. On the other hand, the addition of osmotically pre-treated Lyo apple powder (sample 4, 5, 6) causes a statistically significantly increase in Ca, K, and Na in muffin samples compared with control sample 1. These data confirm that OT+Lyo apple powder is a rich source of minerals, and that its addition to muffins significantly improves mineral content of intake. The highest values of Ca, K, and Na (56.07 mg/100 g; 256.85 mg/100 g; 43.0 mg/100 g, respectively) were found in the muffin sample with 30% OT+Lyo apple powder (sample 7). Sugar beet molasses is rich in essential micronutrients, including minerals (especially K, 4090.0 mg/100g, Ca, 201.1 mg/100 g, and Na, 572.0 mg/100 g), vitamins, glutamic acid, organic acids, pectin, etc. [43,44]. By using this side product of the sugar industry as an osmotic medium for the preparation of apple powder, the nutrient properties of muffins can be improved for maintaining health and ensuring optimal body function [45,46].

The technological characteristics of muffins with Lyo apple and OT+Lyo apple powder are shown in Table 4. The percentage addition of Lyo apple powder (10 to 30%) and osmotically pre-treated Lyo apple powder (10 to 30%) affects the slight reduction in the specific volume of the finished product (samples 1–3 and samples 4–6), although there are no statistically significant differences between samples. Hence, these data indicate that the addition of Lyo apple powder does not disturb the structure of the finished product to any great extent. The partial substitution of flour with the Lyo apple and osmotically pre-treated apple powder did not affect specific weight while affecting the muffin’s texture. For muffin samples 2–4, a percentage increase in the amount of Lyo apple powder results in a decrease in hardness; the addition of Lyo apple powder also increases the springiness of the muffins compared to the control sample (1).

The hardness of a muffin sample is expressed as the maximum peak force at the first bite, and depends mainly on moisture and fat content [47]. By increasing the quantity of Lyo apple powder (10, 20, and 30%) in muffin dough (sample 2–4), hardness statistically increased (*p* ≤ 0.05) compared with the control sample (1). Conversely, an increase in the quantity of Lyo+OT apple powder added to muffins (10, 20, and 30%) leads to a statistically significant decrease in hardness (compared between samples 5–7).

Osmotic dehydration cause changes to the cell walls of the apple itself, which is reflected in significantly lower hardness of the muffin (samples 5–7) compared to the control sample (1) and compared to the samples with the apple powder (2–4). The springiness of the muffins refers to the rate of deformation recovery at which a deformed muffin sample shifts back to its undeformed shape after the involved deforming tension is withdrawn [43]. With a percentage increase in apple and osmotically dehydrated apple powder content, there is an increase in springiness compared to the control sample (1), except for sample 7. Sample 7 (muffin with 30% OT+Lyo apple powder) has the lowest value of hardness and springiness.

As one of the crucial factors for consumers’ acceptance of a product, color is a primary concern in valuing consumers’ opinions, quality features, and acceptability of a food product [47]. The addition of Lyo apple powder (samples 2–4) in the amount of 10 to 30% leads to a statistically significant increase in samples’ lightness compared to the control sample (1), while the addition of osmotically pre-treated apple powder affects the lightness of the muffin samples oppositely (samples 5–7) (Table 5). Based on instrumental data on muffin color, it is observed that muffin samples with osmotically pre-treated apple powder (5–7) have statistically significant lower lightness compared to control sample 1 and compared to muffins with Lyo apple powder addition (2–4). These data indicate that the addition of OT+Lyo apple powder contributes to darkness in the finished product, probably as a result of the impact of the dark-coloured molasses via solid gain in OT+Lyo samples. Increasing the amount of Lyo apple and osmotically pre-treated apple powder (from 10 to 30%) in the muffin governs an augmentation in the share of red tone in the control sample (1). Differences in redness of muffins with Lyo apple and osmotically dehydrated+Lyo apple powder range from 9.39 to 13.14; the differences are not significantly pronounced between samples 3–4 and samples 4–7. According to the measured data (Table 5), it can be seen that muffins with Lyo apple powder (samples 2–4) have a higher proportion of yellow tone compared to the control sample (1) as well as samples 5–7. Higher values of OT+Lyo apple powder addition (10, 20, and 30%) in the muffin samples results in a decrease in yellow tone (samples 5, 6, 7). The addition of Lyo apple powder (samples 2–4) affects the increase in whiteness parameters in relation to the control sample (1). Samples of muffins with osmotically dehydrated apple powder (5–7) have significantly lower whiteness compared to muffins with apple (samples 2–4) as well as the control sample (1). Obtained results point to the fact that osmotic dehydration in sugar beet molasses affects the decrease in whiteness of the apple and thus the finished product, based on the color matters achieved from sugar beet molasses which reveals the solid gain of osmodehydrated apple. Šobot et al. 2019 [48] recorded the same trend in changing the whiteness of biscuits by adding osmotically dehydrated wild garlic in sugar beet molasses. Alteration of the natural color of apples after osmotic treatment in sugar beet molasses is apparent; however, using this apple powder as a supplement in bakery products could positively impact their color (natural light brown color) [49]. Kim et al. 2020 [50] stated that in terms of the texture and chromaticity of muffins formulated with rising amounts of ‘Fuji’ apple powder, the hardness and L* and b* values declined, while the cohesiveness and the a* value showed the opposite trend. The highest values of ΔE response were obtained for samples 4 and 7, indicating the highest colour variation between control and observed samples.

### 3.1. Correlation between Observed Chemical Composition and Technological Characteristic Responses and Addition of Apple Powder to the Spelt Muffin Formulation

**Figure 2:**
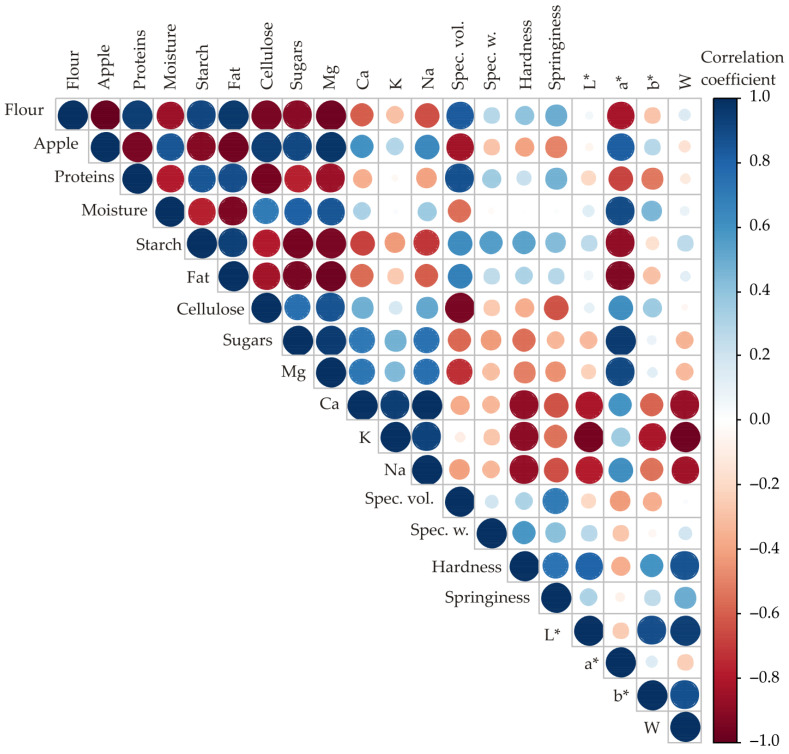
Color correlation diagram between all tested responses of muffins with additions of apple and OT apple powder.

Figure 2 illustrates a color correlation graph for all observed responses of muffin samples’ nutritional and technological quality attributes. Values of correlation coefficients between two tested responses are illustrated by color (blue for positive and red for negative correlation) and by the dimensions of the circles. There is a high positive correlation among the following responses: apple powder addition, moisture, sugar, cellulose, mineral matter content responses; a* and b* of instrumental color responses. On the other hand, a highly negative correlation can be seen among the following responses: proteins, starch, fat of chemical content; all technological quality parameters; L* and W of instrumental color responses. The flour content is positively correlated to the contents of proteins, starch, and fat (correlation coefficient r = 0.944, statistically significant at *p* ≤ 0.01; r = 0.919, *p* ≤ 0.01 and r = 0.966, *p* ≤ 0.01, respectively), and negatively correlated to the contents of cellulose and sugars (r = −0. 946, *p* ≤ 0.01 and r = −0.905, *p* ≤ 0.01, respectively). Furthermore, the flour content is positively correlated to the specific volume (r = 0.831, *p* ≤ 0.05), and negatively correlated to moisture content and colour coordinate a* (r = 0.−852, *p* ≤ 0.05 and r = −0.827, *p* ≤ 0.05, respectively). The apple content is correlated negatively to flour content.

The protein content is positively correlated to starch and fat content, and to specific volume (r = 0.852, statistically significant at *p* ≤ 0.05; r = 0.888, *p* ≤ 0.01, respectively), and also negatively correlated to the content of moisture, sugars, and cellulose (r = −0.791, *p* ≤ 0.05; r = −0.770, *p* ≤ 0.05; and r = −0.057 *p* ≤ 0.01).

The starch content is negatively correlated to fat content (r = −0.776, *p* ≤ 0.05).

The fat content is negatively correlated to moisture (r = −0.939, *p* ≤ 0.01) and positively correlated to fat content (r = 0.943, *p* ≤ 0.01).

The cellulose content is negatively correlated to starch and fat content (r = −0.797, *p* ≤ 0.05 and r = −0.839, *p* ≤ 0.05, respectively).

The sugar content is positively correlated to moisture content (r = 0.817, *p* ≤ 0.05) and negatively correlated to the content of starch and fat (r = −0.955, *p* ≤ 0.01 and r = −0.940, *p* ≤ 0.01, respectively).

The content of K is positively correlated to the content of Mg and Ca (r = 0.850, *p* ≤ 0.05 and r = 0.940, *p* ≤ 0.01, respectively), while the content of Na is positively correlated to the content of Ca and K (r = 0.999, *p* ≤ 0.01 and r = 0.924, *p* ≤ 0.01, respectively).

The specific volume is negatively correlated to cellulose content (r = −0.946, *p* ≤ 0.01).

The hardness of muffin samples was negatively correlated to the contents of Ca, K, and Na (r = −0.887, *p* ≤ 0.01; r = −0.891, *p* ≤ 0.01; and r = −0.878, *p* ≤ 0.01, respectively).

The colour coordinate L* of muffin samples was negatively correlated to the contents of Mg, Ca, K, and Na (r = −0.929, *p* ≤ 0.01; r = −0.813, *p* ≤ 0.05; r = −0.950, *p* ≤ 0.01; and r = −0.786, *p* ≤ 0.05, respectively) and positively correlated to the hardness of muffin samples (r = 0.802, *p* ≤ 0.05).

The colour coordinate a* of muffin samples was negatively correlated to the contents of starch and fat (r = −0.883, *p* ≤ 0.01 and r = −0.928, *p* ≤ 0.01, respectively) and positively correlated to moisture and sugar content (r = 0.895, *p* ≤ 0.01 and r = 0.958, *p* ≤ 0.01, respectively).

The colour coordinate b* of muffin samples was negatively correlated to the contents of Mg and K (r = −0.983, *p* ≤ 0.01 and r = −0.817, *p* ≤ 0.05, respectively) and positively correlated to the colour coordinate L* of muffin samples (r = 0.889, *p* ≤ 0.01).

The colour coordinate W of muffin samples was negatively correlated to the contents of Mg, Ca, K, and Na (r = −0.908, *p* ≤ 0.01; r = −0.865, *p* ≤ 0.05; r = −0.971, *p* ≤ 0.01; and r = −0.842, *p* ≤ 0.05, respectively) and positively correlated to the colour coordinates L* and b* of muffin samples (r = 0.943, *p* ≤ 0.01 and r = 0.879, *p* ≤ 0.01, respectively).

Color correlation analysis confirmed that apple powder (especially osmotically treated) added to the muffin dough positively affected the majority of the chemical composition responses while negatively affecting technological characteristic responses. The incorporation of nutritively rich and also cellulosic material consequently provided a negative effect on the finished product’s total quality. Similar results were reported by Filipovic et al. [51], where the addition of dehydrated peach (especially with osmotic pre-treatment) to cookie dough positively affected most of the nutritive quality responses, while technological responses generally were negatively correlated.

### 3.2. Principal Component Analysis

**Figure 3:**
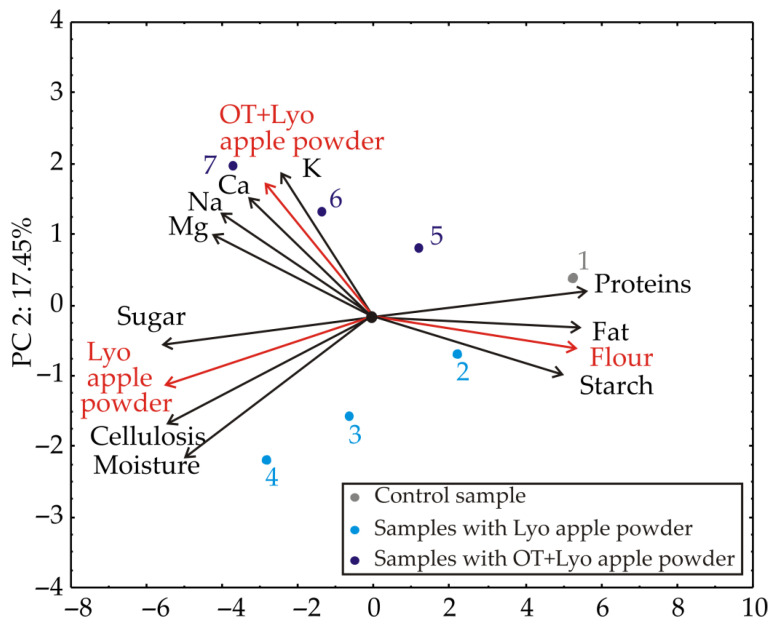
PCA biplot diagram depicting the relationships among muffin samples regarding chemical composition.

**Figure 4:**
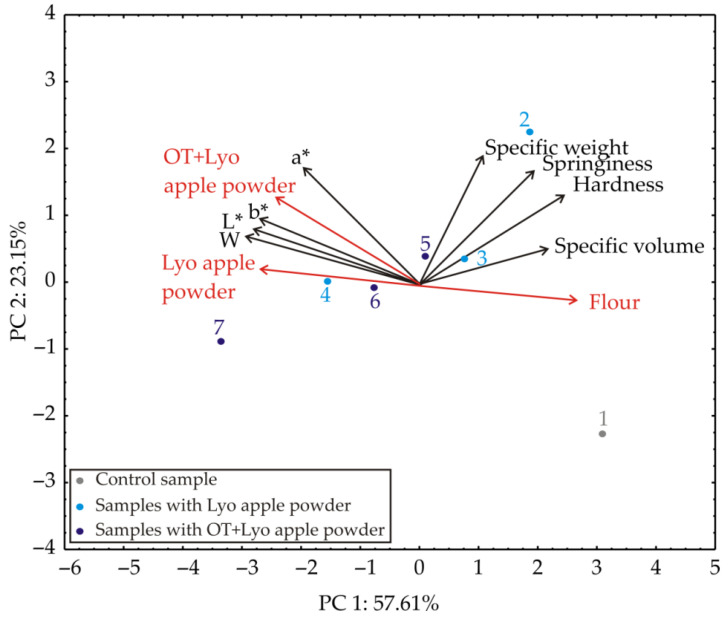
PCA biplot diagram depicting the relationships among muffin samples regarding color and technological characteristics.

In order to adequately describe the configuration of the results that would provide insight to the appearance of the muffin samples, PCA was exploited, and the results are exposed in Figure 3 (regarding chemical composition) and Figure 4 (regarding color and technological characteristics). Taking into account the graph of the PCA conducted on the data, the first two PCs clarified 91.72% of the total variance in the experimental data. The factors’ projection indicated that the content of the flour, apple powder, moisture, proteins, starch, oil, cellulose, and sugar mainly contributed to the first principal component, PC1 (10.05%, 10.05%, 9.45%, 7.65%, 8.12%, 9.38%, 9.45%, 8.07%, and 9.49%, respectively). In comparison, Ca, K, and Na content contributed more to the second principal component, PC2 (22.89%, 36.70%, and 20.84%, respectively).

The separation among control and samples containing Lyo apple and OT+Lyo apple powder could be discovered from the PCA biplot, Figure 3. The most pronounced K, Ca, Mg, and Na content was identified for the sample with the highest amount of OT+Lyo apple powder (sample 7). The map of the PCA analysis revealed that the first principal component represented the differentiation between the samples according to flour and apple powder content. In contrast, the second principal component described the variations in chemical composition between samples.

The first two PCs outlined 80.76% of the total variance in the experimental data. The factors’ projection suggested that specific volume, L*, b*, and W mainly contributed to the first principal component, PC1 (10.34%, 18.14%, 18.91%, and 18.77%, respectively). The specific weight, hardness, and springiness contributed more to the second principal component, PC2 (27.98%, 15.98%, 22.56%, and 21.66%, respectively). The differentiation between samples could be recognized from the PCA graphic, where the samples with the higher amount of apple powder are arranged on the left side of the graphic. In comparison, the samples with a lower amount of apple powder are arranged on the right side of the graphic.

### 3.3. ANN Model and Global Sensitivity Analysis—Yoon’s Interpretation Method

The substitution of flour with the Lyo apple powder and OT+Lyo apple powder on the chemical composition, technological characteristics, and color of the muffins was examined using an ANN model. The acquired optimal neural network model expressed an acceptable generalization capability for the experimental data, and could correctly anticipate the output parameters of the muffin samples for a range of the observed input parameters. In conformity with the ANN performance, the optimal number of neurons in the hidden layer for the chemical composition, technological characteristics, and color parameters calculation was 7 (network MLP 5-7-18), in order to attain elevated values of coefficient of determination, r^2^ (overall 0.998 for ANN throughout the training period), and depleted values of the sum of squares (SOS). The applied training algorithm was BFGS 274, with a hyperbolic tangent for hidden activation and identity for output activation function. The ANN model was complex, with 179 weight biases for observed muffin samples. In order to comprehensively describe the impact of input variables on the ascertained output variables, the addition of flour and apple powder and treatment of the muffins’ chemical composition and technological parameters were analysed using Yoon’s global sensitivity equation in line with the weight coefficients of the developed ANN model [10,52].

**Figure 5:**
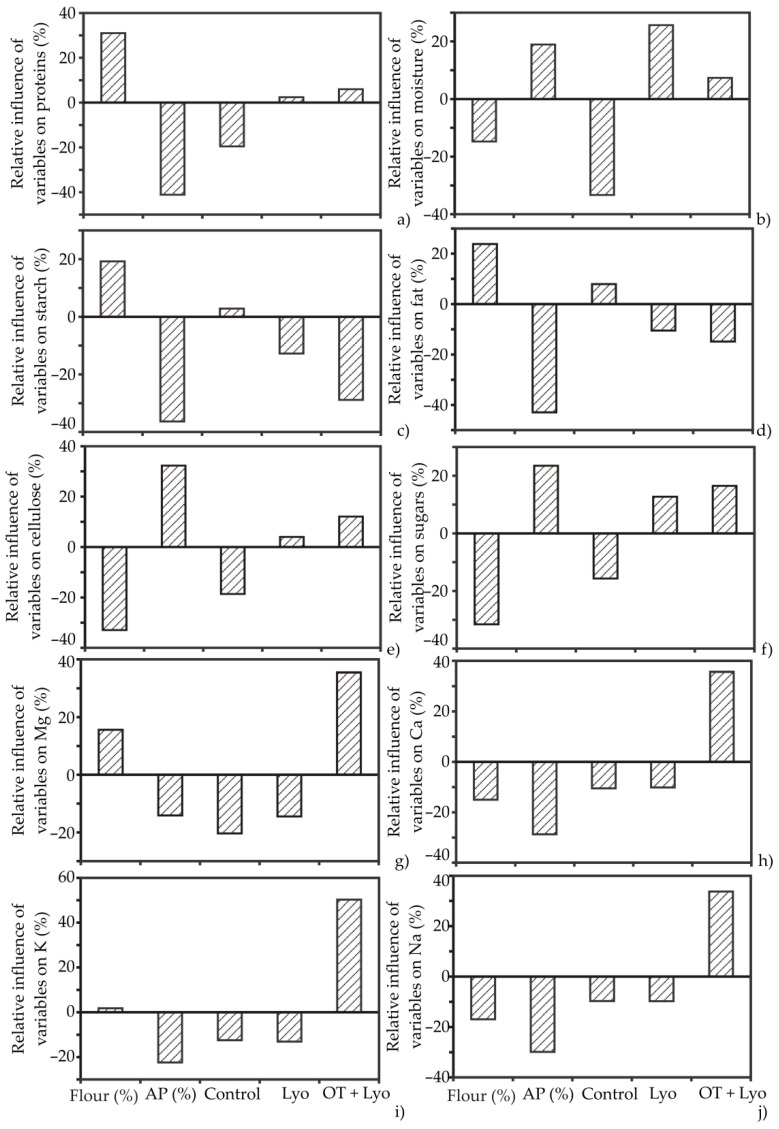
The relative importance of the content of flour, apple powder, and applied treatment on (**a**) proteins; (**b**) moisture; (**c**) starch; (**d**) fat; (**e**) cellulose; (**f**) sugars; (**g**) Mg; (**h**) Ca; (**i**) K; and (**j**) Na, determined using the Yoon interpretation method.

**Figure 6:**
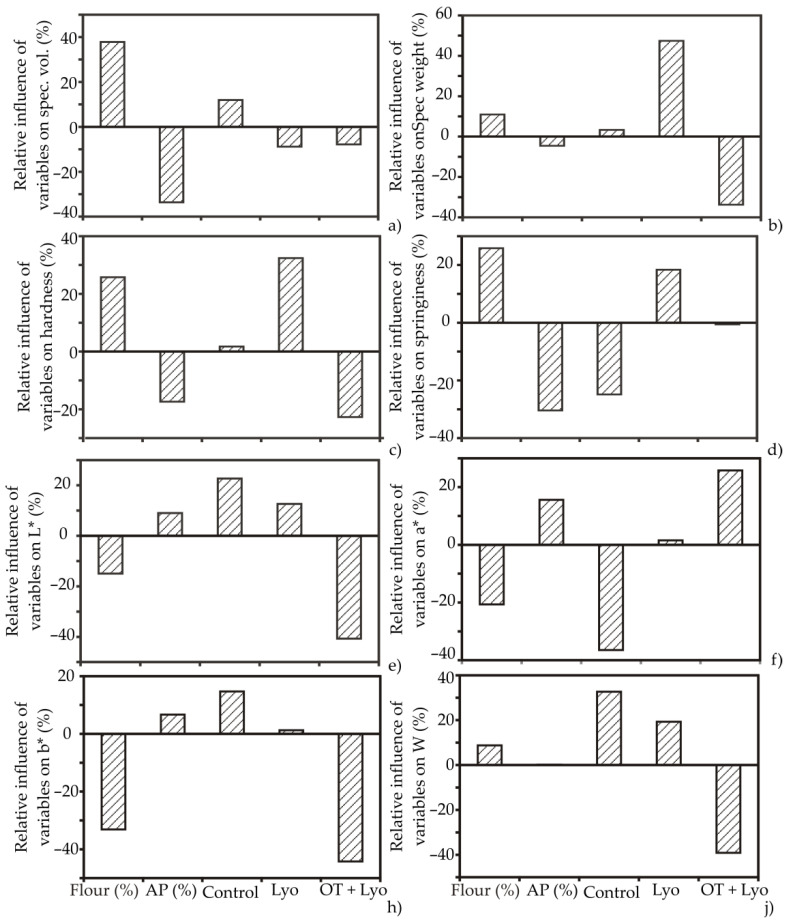
The relative importance of the content of flour, apple powder, and applied treatment on (**a**) specific volume; (**b**) specific weight; (**c**) hardness; (**d**) springiness; (**e**) L*; (**f**) a*; (**h**) b* and (**j**) W, determined using the Yoon interpretation method.

Following the Yoon presentation method of a declared ANN model, the graphical appearance of the ANN model results is displayed in Figure 5 and Figure 6, respectively. Based on Figure 5b–f, the addition of apple powder and osmotic pre-treatment positively influenced the moisture content (relative influences were 19.3% and 27.2%, respectively), cellulose (31.4% and 16.12%), and sugars (22.18% and 14.22%) in the muffin samples, while the impact on fat (−41.21% and −17.20%) and starch (−38.20% and 27.72%) was negative. The most influential parameter negatively affecting protein content in muffin samples was the addition of the apple powder with an approximate relative importance of −40%; meanwhile, the impact of flour addition was the opposite, at 30.11%. Osmotic pre-treatment in sugar beet molasses followed by lyophilisation to obtain apple powder was the most positively influential parameter among the observed minerals in the muffin samples, with relative influences of 37.11% for Mg, 38.33% for Ca, and 33.34% for Na. However, the highest value of 52.78% was observed for K (Figure 5g–j). Quite the opposite trend was noticed for Mg, Ca, K, and Na content, with the addition of lyophilized apple powder without osmotic dehydration treatment, and also for the control sample.

According to Figure 6, the addition of the apple powder previously osmotically treated in sugar beet molasses negatively influenced all observed technological parameters of the muffin samples with approximate relative influences of −7.77% for specific volume, −36.72% for specific weight, −24.66% for hardness, and −2.22% for springiness. The same trend was noticed for color parameters −40.13% for L*, −44.23% for b*, and −39.85% for W, while the influence on a* was quite the opposite (27.77%). On the other hand, the addition of apple powder without the osmotic pre-treatment positively influenced all experimental parameters (with approximate relative influences of 48.82%, 33.77%, 18.86%, 13.72%, 2.81%, 2.11%, and 19.98% for specific weight, hardness, springiness, L*, a*, b* and W, respectively), except for specific volume (−4.98%).

### 3.4. Correspondence Analysis, Sensory Evaluation of Muffins, and Consumer Acceptance Test

In Table 6, four descriptive sensory analysis responses for muffin samples with and without the addition of Lyo and OT+Lyo apple powder are shown.

In Table 7, the consumer acceptance test results of the muffin samples with and without the addition of Lyo and OT+Lyo apple powder are shown.

**Figure 7:**
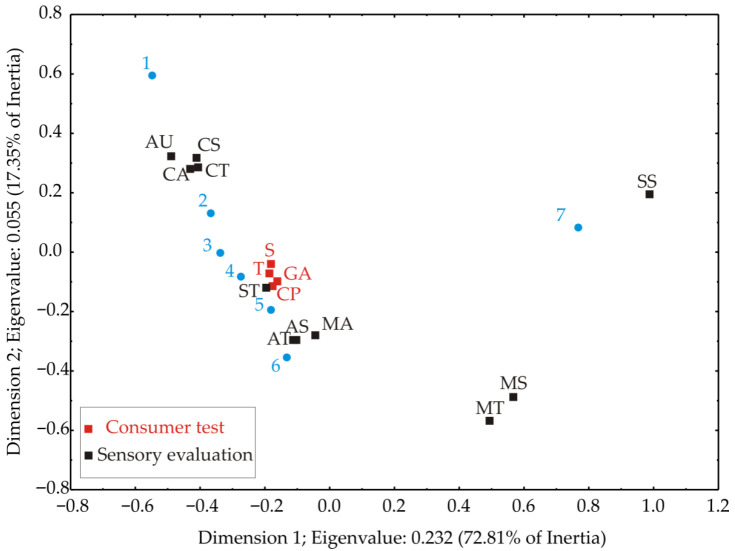
Sensory evaluation and consumer acceptance test of the muffins. ST—sweet taste, MT—molasses taste, AT—apple taste, CT—characteristic taste, AS—apple smell, CS—characteristic smell, SS—sweet smell, MS—molasses smell, CA—characteristic appearance, AU—appearance of upper surface, MA—mastication, S—smell, T—taste, GA—general acceptability, and CP—chewing properties.

The graphical appearance of correspondence analysis for experimental results exhibited in Table 6 and Table 7 is illustrated in Figure 7. Significant correspondence was ascertained amid the explored properties (total inertia was 0.318; χ^2^ was 145.26; df = 84; *p* < 0.00004). The first two dimensions explained 90.16% of the total inertia, utilizing a substantially satisfactory quota of the raw information.

Figure 7 shows differentiation in the muffin sensory evaluation and the consumer acceptance test results.

From Figure 7 and Table 6 it can be seen that the descriptors for the smell and taste of muffins with the increase of Lyo apple powder (samples 2–4) and osmotically pre-treated apple powder percentage (samples 5–7) deviate from the characteristic taste and smell concerning muffin sample 1, with the taste and smell of apples and sweets increasing (samples 4 and 7). The addition of apple powder affects the appearance of the product, i.e., it reduced one grade of the descriptor, while the osmotically dehydrated apple led to a reduction of two grades. However, the product still received a “good appearance” grade. The control sample (1) had the lowest mastication score. This result indicates that the raw material composition should be improved. This fact is confirmed by the consumer test (Table 5), where consumers liked sample 1 the least because it obtained the lowest ratings. Sample 4 received the highest rating for mastication (4.83), nearly achieving “excellent quality”, followed by sample 7 with a “very good” rating of 4 for mastication.

According to Figure 7 and Table 7, consumers liked the control sample the least, i.e., a muffin without added apples and osmotically dehydrated apples, in terms of the examined parameters. Taste is among the leading determinants of food preference for consumers [53]. The highest score in the consumer test was given to sample 7, with 30% osmotically dehydrated apple. Consumers liked sample 7 the most. It received grades of 7 (“Liked it moderately”) for taste, smell, and chewing properties, and a grade of 8 (“Liked it very much”) for general acceptability. Table 5 shows that adding 30% apple (sample 4) positively affected taste, smell, chewing properties, and general acceptability, since sample 4, and then sample 7, were best evaluated by consumers. Based on the results of the consumer test, it can be seen that consumers clearly preferred muffins with the addition of apples (samples 2–4) and osmotically dehydrated apples (5–7), as far as all the observed parameters to the control muffin (sample 1) were concerned.

## 4. Conclusions

The results revealed that spelt muffins with the addition of osmotically pre-treated apple powder were nutritionally enriched compared to the control muffins. Furthermore, by comparing the chemical composition of muffin samples, it was observed that the addition of osmotically pre-treated apple powder increases moisture, cellulose, and sugar content, as well as Ca, K, and Na content as a consequence of the mass transfer from sugar beet molasses. These results open an innovative aspect in which an agro-industrial side product, such as sugar beet molasses, could be utilized as a promising osmotic solution with the possibility of raising the nutritional properties of baked products.

The percentage addition of apple powder (10 to 30%) and osmotically treated apple powder affects the reduction in the specific volume of the finished product without affecting specific weight. Moreover, it alters the muffin’s textural attributes by decreasing the muffin samples’ hardness and springiness.

Yoon’s interpretation method performed on a created ANN model recognized the addition of osmotically pre-treated apple powder and osmotic treatment as the most influential parameters that affected the observed parameters.

Based on sensory evaluation of the muffins, percentage increases in apple powder and osmotically treated apple powder positively affect taste and smell, while slightly reducing the appearance grade of the product.

According to the consumer test results, the sample with the highest content of osmotically dehydrated apple was the one that achieved the highest scores. It can be concluded that the addition of apple powder osmotically pre-treated in sugar beet molasses to muffin-like bakery products as a flour supplement could be recommended as a valuable ingredient due to its nutritional and sensory properties.

## Figures and Tables

**Table 1:** Muffin samples’ compositions.

No.	Sample	Flour Content (%)	Lyo Apple or OT+Lyo Apple Powder Content (%)
1	Control	100	0
2	10% Lyo apple powder	90	10
3	20% Lyo apple powder	80	20
4	30% Lyo apple powder	70	30
5	10% OT+Lyo apple powder	90	10
6	20% OT+Lyo apple powder	80	20
7	30% OT+Lyo apple powder	70	30

**Table 2:** Chemical composition of muffins with Lyo apple and OT+Lyo apple powder.

No.	Proteins (% d.m)	Moisture (%)	Starch (% d.m)	Fat (% d.m)	Cellulose (% d.m)	Sugars (% d.m)
1	12.75 ± 0.05 ^a^	9.59 ± 0.13 ^a^	33.59 ± 0.66 ^d^	14.40 ± 0.91 ^b^	0.49 ± 0.08 ^a^	31.30 ± 0.66 ^a^
2	12.48 ± 0.16 ^a^	11.30 ± 0.21 ^b^	32.91 ± 0.54 ^d^	13.24 ± 0.54 ^a,b^	0.56 ± 0.05 ^a,b^	32.84 ± 0.54 ^a^
3	11.40 ± 0.20 ^b,d^	12.07 ± 0.69 ^b^	26.04 ± 0.39 ^b^	12.27 ± 0.72 ^a,b^	0.67 ± 0.03 ^c^	35.01 ± 0.39 ^b^
4	10.57 ± 0.15 ^c^	12.16 ± 0.57 ^b^	23.81 ± 0.88 ^a^	11.79 ± 0.83 ^a^	0.90 ± 0.09 ^d^	36.20 ± 0.88 ^b,c^
5	12.59 ± 0.35 ^a^	11.15 ± 0.42 ^b^	28.34 ± 0.92 ^c^	13.02 ± 0.89 ^a.b^	0.50 ± 0.03 ^a.b^	35.19 ± 0.92 ^b,c^
6	11.75 ± 0.09 ^b^	11.55 ± 0.44 ^b^	24.36 ± 0.51 ^a^	12.28 ± 1.10 ^a,b^	0.64 ± 0.05 ^b,c^	35.60 ± 0.51 ^b,c^
7	11.20 ± 0.03 ^d^	11.66 ± 0.38 ^b^	23.49 ± 0.47 ^a^	11.94 ± 1.01 ^a^	0.86 ± 0.02 ^d^	36.84 ± 0.47 ^c^

Different letters (^a, b, c, d^) printed the same column show significantly different means of observed data (*p* ≤ 0.05), according to post hoc Tukey’s HSD test.

**Table 3:** The contents of selected minerals in muffins with Lyo apple and OT+Lyo apple powder (mg/100 g).

No.	Mg	Ca	K	Na
1	80.06 ± 0.66 ^e^	49.73 ± 0.66 ^a^	204.43 ± 0.66 ^d^	34.70 ± 0.66 ^a^
2	75.08 ± 0.54 ^d^	50.14 ± 0.54 ^a^	200.11 ± 0.54 ^c^	35.35 ± 0.54 ^a.b^
3	70.09 ± 0.39 ^c^	50.54 ± 0.39 ^a,b^	195.79 ± 0.39 ^b^	36.01 ± 0.39 ^a,b,c^
4	65.12 ± 0.88 ^a^	50.94 ± 0.88 ^a,b^	191.47 ± 0.88 ^a^	36.66 ± 0.88 ^b,c^
5	81.83 ± 0.92 ^d^	51.85 ± 0.92 ^b^	221.91 ± 0.92 ^e^	37.47 ± 0.92 ^c^
6	82.60 ± 0.51 ^c^	53.96 ± 0.51 ^c^	239.38 ± 0.51 ^f^	40.23 ± 0.51 ^d^
7	85.37 ± 0.47 ^b^	56.07 ± 0.47 ^d^	256.85 ± 0.47 ^g^	43.00 ± 0.47 ^e^

Different letters (^a, b, c, d, e, f, g^) within the same column show significantly different means of observed data (*p* ≤ 0.05), according to post hoc Tukey’s HSD test.

**Table 4:** Technological characteristics of muffins with Lyo apple and OT+Lyo apple powder.

No.	Spec Volume (mL)	Spec Weight (g)	Hardness (g)	Springiness (%)
1	1.78 ± 0.18 ^a^	40.00 ± 0.66 ^a^	1383.66 ± 0.66 ^e^	56.83 ± 0.66 ^b^
2	1.77 ± 0.24 ^a^	41.00 ± 0.54 ^a^	1785.18 ± 0.54 ^g^	60.01 ± 0.54 ^d^
3	1.73 ± 0.16 ^a^	40.00 ± 0.39 ^a^	1585.57 ± 0.39 ^f^	59.11 ± 0.39 ^c,d^
4	1.68 ± 0.10 ^a^	40.00 ± 0.88 ^a^	1465.28 ± 0.88 ^d^	57.09 ± 0.88 ^b^
5	1.81 ± 0.27 ^a^	40.00 ± 0.92 ^a^	1229.96 ± 0.92 ^c^	60.30 ± 0.92 ^d^
6	1.78 ± 0.09 ^a^	40.00 ± 0.51 ^a^	1205.68 ± 0.51 ^b^	57.87 ± 0.51 ^b,c^
7	1.67 ± 0.14 ^a^	40.00 ± 0.47 ^a^	737.26 ± 0.47 ^a^	53.63 ± 0.47 ^a^

Different letters (^a, b, c, d, e, f, g^) within the same column show significantly different means of observed data (*p* ≤ 0.05), according to post hoc Tukey’s HSD test.

**Table 5:** Color of muffins with Lyo apple and OT+Lyo apple.

No.	L*	a*	b*	ΔE	W
1	47.17 ± 0.54 ^c^	5.45 ± 0.66 ^a^	26.85 ± 0.66 ^c,d^	-	21.19 ± 0.88 ^c^
2	49.42 ± 0.66 ^d^	9.39 ± 0.54 ^b^	27.95 ± 0.54 ^d^	4.67 ± 0.05 ^a^	21.62 ± 0.39 ^c^
3	50.95 ± 0.39 ^d^	12.44 ± 0.88 ^c^	33.58 ± 0.39 ^e^	10.41 ± 0.07 ^b^	22.39 ± 0.54 ^c,d^
4	53.69 ± 0.88 ^e^	12.82 ± 0.39 ^c^	36.53 ± 0.88 ^f^	13.80 ± 0.17 ^c^	23.84 ± 0.66 ^d^
5	40.55 ± 0.92 ^b^	12.46 ± 0.51 ^c^	26.22 ± 0.92 ^b,c^	9.66 ± 0.14 ^a^	19.20 ± 0.92 ^b^
6	38.16 ± 0.47 ^a^	12.56 ± 0.92 ^c^	24.85 ± 0.51 ^a,b^	11.65 ± 0.22 ^b^	18.88 ± 0.51 ^b^
7	37.53 ± 0.51 ^a^	13.14 ± 0.47 ^c^	23.39 ± 0.47 ^a^	12.81 ± 0.30 ^c^	15.80 ± 0.47 ^a^

Different letters (^a, b, c, d, e, f^) within the same column show significantly different means of observed data (*p* ≤ 0.05), according to post hoc Tukey’s HSD test.

**Table 6:** Sensory evaluation of the muffins.

No.	Smell				Taste				Appearance		Mastication
	Characteristic	Apple	Sweet	Molasses	Characteristic	Apple	Sweet	Molasses	Characteristic	The Appearance of the Upper Surface	
1	5.0 ± 0.0 ^d^	0	1.67 ± 0.52 ^a^	0	5.0 ± 0.0 ^b^	0	1.83 ± 0.41 ^a^	0	5.00 ± 0.0 ^b^	4.83 ± 0.41 ^c^	0.33 ± 0.52 ^a^
2	4.0 ± 0.63 ^c^	3.00 ± 0.63 ^a^	3.33 ± 0.52 ^b^	0	4.17 ± 0.41 ^a,b^	3.17 ± 0.41 ^a^	3.83 ± 0.41 ^b^	0	4.33 ± 0.52 ^a,b^	4.33 ± 0.52 ^b,c^	1.17 ± 0.41 ^a,b^
3	3.67 ± 0.52 ^b^	3.83 ± 0.41 ^a,b^	3.33 ± 0.52 ^b^	0	3.83 ± 0.75 ^a,b^	4.17 ± 0.41 ^b,c^	4.00 ± 0.63 ^b^	0	4.00 ± 0.63 ^a,b^	3.67 ± 1.03 ^a,b,c^	2.33 ± 0.52 ^b,c^
4	4.17 ± 0.75 ^c^	4.83 ± 0.41 ^b^	4.87 ± 0.52 ^b^	0	4.33 ± 0.75 ^a,b^	5.00 ± 0.0 ^c^	4.87 ± 0.52 ^b^	0	3.83 ± 0.41 ^a,b^	3.17 ± 0.41 ^a,b^	4.83 ± 0.41 ^e^
5	2.67 ± 0.52 ^a^	3.00 ± 0.63 ^a^	4.00 ± 0.63 ^a,b^	2.17 ± 0.75 ^a^	3.17 ± 0.41 ^a^	3.33 ± 0.41 ^a,b^	4.33 ± 0.52 ^b^	2.33 ± 0.52 ^a^	3.33 ± 0.52 ^a^	3.0 ± 0.63 ^a,b^	1.33 ± 0.52 ^a,b^
6	2.87 ± 0.75 ^a,b^	4.17 ± 0.41 ^a,b^	3.83 ± 0.41 ^a,b^	3.00 ± 0.89 ^a^	3.00 ± 0.63 ^a^	4.33 ± 0.52 ^c^	4.50 ± 0.50 ^b^	4.00 ± 0.63 ^b^	3.17 ± 0.75 ^a^	2.83 ± 0.41 ^a,b^	3.33 ± 0.52 ^c,d^
7	3.17 ± 0.75 ^b^	4.83 ± 0.41 ^b^	54.83 ± 0.41 ^d^	4.50 ± 0.55 ^b^	3.33 ± 0.52 ^a^	5.00 ± 0.0 ^c^	5.00 ± 0.0 ^b^	4.67 ± 0.52 ^b^	3.0 ± 0.63 ^a^	2.17 ± 0.41 ^a^	4.00 ± 0.63 ^d,e^

Different letters (^a, b, c, d, e^) within the same column show significantly different means of observed data (*p* ≤ 0.05), according to post hoc Tukey’s HSD test.

**Table 7:** Consumer acceptance test results of the muffins.

No.	Taste	Smell	Chewing Properties	General Acceptability
1	3.25 ± 1.5 ^a^	3.5 ± 1.29 ^a^	2.75 ± 1.26 ^a^	3 ± 1.41 ^a^
2	5.25 ± 1.26 ^a,b^	5.0 ± 1.41 ^a^	5.0 ± 1.41 ^a,b^	5.0 ± 1.5 ^a,b^
3	5.5 ± 2.08 ^a,b^	5.25 ± 2.06 ^a^	6.0 ± 1.82 ^a,b^	5.75 ± 2.22 ^a,b^
4	6.25 ± 1.71 ^a,b^	6.25 ± 2.21 ^a^	7.25 ± 0.96 ^b^	6.5 ± 1.74 ^a,b^
5	6.25 ± 0.5 ^a,b^	5.75 ± 0.96 ^a^	6.25 ± 0.96 ^b^	6.5 ± 0.58 ^a,b^
6	6.25 ± 0.96 ^a,b^	6.0 ± 0.82 ^a^	6.5 ± 1.29 ^b^	6.5 ± 1.0 ^a,b^
7	7.5 ± 1.29 ^b^	7.5 ± 1.73 ^a^	7.75 ± 1.26 ^b^	8.0 ± 1.41 ^b^

Different letters (^a, b^) within the same column show significantly different means of observed data (*p* ≤ 0.05), according to post hoc Tukey’s HSD test.

## Data Availability

Data are contained within the article.
